# Toll-Like Receptor 2 is Involved in Abnormal Pregnancy in Mice Infected with *Toxoplasma gondii* During Late Pregnancy

**DOI:** 10.3389/fmicb.2021.741104

**Published:** 2021-10-05

**Authors:** Rina Ikeda, Nanako Ushio, Ahmed M. Abdou, Hidefumi Furuoka, Yoshifumi Nishikawa

**Affiliations:** ^1^National Research Center for Protozoan Diseases, Obihiro University of Agriculture and Veterinary Medicine, Obihiro, Japan; ^2^Department of Forensic Medicine and Toxicology, Faculty of Veterinary Medicine, South Valley University, Qena, Egypt; ^3^Division of Pathobiological Science, Department of Basic Veterinary Medicine, Obihiro University of Agriculture and Veterinary Medicine, Obihiro, Japan

**Keywords:** *Toxoplasma gondii*, congenital toxoplasmosis, TLR2, mouse, multiple calcifications, pregnancy

## Abstract

Infection with *Toxoplasma gondii* during pregnancy causes failure of pregnancy maintenance, resulting in fetal death, abortion, stillbirth, or premature birth, but the mechanism of disease onset remains unclear. Although Toll-like receptor 2 (TLR2) is expressed on antigen-presenting cells and trophoblasts, the role of TLR2 in *T. gondii* infection during pregnancy is unknown. In this study, we investigated the role of TLR2 in congenital toxoplasmosis using TLR2-deficient (TLR2^−/−^) mice. *T. gondii* infection on gestational day 12.5 (Gd12.5) induced more abnormal pregnancy, including premature birth and stillbirth, in wild-type mice than in TLR2^−/−^ mice. Multiple calcifications were observed in the placentas of the infected wild-type mice. At Gd18.5 (6days postinfection), the parasite numbers in the placenta and uterus and the histological changes did not differ significantly between the wild-type and TLR2^−/−^ mice. However, *T. gondii* infection reduced the mRNA expression of interleukin-12p40 (IL-12p40) and increased IL-4 and IL-10 mRNAs in the placentas of the wild-type mice. In contrast, the placentas of the TLR2^−/−^ mice showed no changes in the expression of these cytokines, including IL-6 and tumor necrosis factor α, in response to *T. gondii* infection. Serum interferon-γ levels were significantly lower in the infected TLR2^−/−^ mice than in the infected wild-type mice on Gd18.5. Thus, the TLR2^−/−^ mice were less susceptible to the induction of immune responses by *T. gondii* infection during late pregnancy. Therefore, TLR2 signaling may play a role in the development of disease states during pregnancy, specifically placental hypofunction.

## Introduction

*Toxoplasma gondii* is an obligate intracellular eukaryotic parasite of the phylum Apicomplexa, which is thought to infect about 30% of the world’s population (Montoya and Liesenfeld, 2004). It infects humans and homothermic animals, causing toxoplasmosis. In humans, first infection with *T. gondii* during pregnancy can cause a wide variety of morbidities, including abortion, stillbirth, and premature birth, depending upon the stage of pregnancy (Hill and Dubey, 2002; Givens and Marley, 2008). The development of preventive and therapeutic measures against *T. gondii* infections during pregnancy is an important issue, but the mechanism of disease onset remains unclear.

The dynamics of the immune system during pregnancy are associated with the maintenance of pregnancy. Pathogen infection during early pregnancy stimulates inflammatory responses at the fetal–maternal interface, resulting in embryo resorption and abortion (Entrican, 2002). During *T. gondii* infection, the proinflammatory T-helper 1 (Th1)-type cytokines, such as tumor necrosis factor α (TNF-α), interferon γ (IFN-γ), interleukin 2 (IL2) and migration inhibitory factor, can be detrimental to pregnancy and fetal survival, whereas such inflammatory responses play an important role in the host defenses (Chaouat et al., 1990; Coutinho et al., 2012; Gomes et al., 2018). In particular, excessive IFN-γ released during pregnancy can cause the apoptosis of mouse decidual cells at the fetal–maternal interface (Senegas et al., 2009; Coutinho et al., 2012) and of human trophoblast cells (Angeloni et al., 2013; Zhang et al., 2015). In previous studies, we showed that chemokine receptors are involved in the host defenses, the apoptosis of embryos, and fetal resorption during early pregnancy. The C-X-C motif chemokine receptor 3 (CXCR3)-dependent immune response reduces the embryo resorption and fetal loss caused by *T. gondii* infection (Nishida et al., 2021), whereas C-C chemokine receptor type 5 triggers the apoptosis of embryos and fetal resorption in mice infected with *T. gondii* (Nishimura et al., 2017). These findings suggest that the maternal immune response is associated with the maintenance or the failure of early pregnancy dependent on the host receptor.

Microbes infect trophoblasts and other placental cells, resulting in pregnancy complications such as abortion and stillbirth (Gibbs, 2002; Goldenberg et al., 2010; Robbins and Bakardjiev, 2012). Placental immune tolerance is required to prevent fetal rejection and maintain pregnancy. The trophoblast expresses pattern recognition receptors (PRRs) and constitutively secretes cytokines to regulate the immune balance at the maternal–fetal borders and to maintain pregnancy. *T. gondii* infection disrupts the balance of PRR-mediated cytokine secretion in the placenta and induces an inflammatory response at the maternal–fetal interface, making the maintenance of pregnancy difficult (Abrahams et al., 2005; Costello et al., 2007). Furthermore, primary trophoblast cells express all the 10 types of TLR, TLR1-10, while choriocarcinoma cell lines possess broad TLR gene expression, but lacked functional cytokine response to TLR ligand activation (Gierman et al., 2015). Toll-like receptor 2 (TLR2) is an important PRR that controls *T. gondii* infection. During *T. gondii* infection in nonpregnant mice, TLR2 induces a Th1 immune response, with the production of IFN-γ, IL-12, and nitric oxide, and plays an important role in the elimination of the parasite (Mun et al., 2003; Umeda et al., 2017; Ihara et al., 2019). TLR2 is also involved in the production of cytokines and prostaglandin E_2_ by brain cells, astrocytes, and microglia (Umeda et al., 2017). It is reported to recognize glycosylphosphatidylinositol from *T. gondii* (Debierre-Grockiego et al., 2003), and is one of the PRRs expressed on antigen-presenting cells and trophoblasts in early and late gestation (Abrahams et al., 2004; Rindsjö et al., 2007). TLR2 tends to be highly expressed in the placentas of pregnant women with chorioamnionitis, suggesting a relationship between TLR2 and pregnancy failure (Kim et al., 2004; Riley and Nelson, 2010; Thaxton et al., 2010).

Significant immunomodulation of the Th1 immune response occurs at mid-gestation, resulting in a Th2 cytokine environment at the maternal–fetal interface (Reinhard et al., 1998). Because *T. gondii* cannot be controlled by Th2 cytokines, the consequence of infection will be the death of the fetus or the offspring may be born and show adverse symptoms at birth (Wang et al., 2011). However, the role of TLR2 in the maintenance of late gestation during *T. gondii* infection remains unknown. In this study, we investigated the disease status of *T. gondii* infection during late gestation using TLR2-deficient (TLR2^−/−^) mice to clarify the TLR2-dependent immunological mechanism of pregnancy failure.

## Materials and Methods

### Ethics Statement

This study was performed in strict accordance with the recommendations of the Guide for the Care and Use of Laboratory Animals of the Ministry of Education, Culture, Sports, Science and Technology, Japan. The protocol was approved by the Committee on the Ethics of Animal Experiments at Obihiro University of Agriculture and Veterinary Medicine, Obihiro, Japan (permit numbers 18–50, 19–56, 20–27). All surgeries were performed under isoflurane anesthesia, and every effort was made to minimize animal suffering.

### Mice

C57BL/6 mice (wild-type) were obtained from CLEA Japan (Tokyo, Japan). Homozygous *TLR2*-knockout (TLR2^−/−^) mice were a kind gift from Dr. Satoshi Uematsu and Dr. Shizuo Akira of Osaka University (Osaka, Japan; Takeuchi et al., 2000). The mice shown in Supplementary Tables S1–S3 were maintained under specific-pathogen-free conditions on a 12-h light/dark cycle with free access to water and food in the animal facility of the National Research Center for Protozoan Diseases at the Obihiro University of Agriculture and Veterinary Medicine.

### Preparation of *T. gondii* Tachyzoites

Kidney epithelial cells extracted from an African green monkey (Vero cells) cultured in Eagle’s minimum essential medium (Sigma, St. Louis, MO, United States) containing 8% heat-inactivated fetal bovine serum and 1% penicillin–streptomycin were used as the host cells for the passage of *T. gondii* (strain PLK; type II) tachyzoites. To purify the tachyzoites, *T. gondii*-infected Vero cells were washed with sterile phosphate-buffered saline detached, suspended in RPMI 1640 medium (Sigma), passed several times through a syringe with a 27G needle, and then passed through a 5.0-mm pore-size filter (Millipore, Bedford, MA, United States).

### Comparison of Abnormal Pregnancies Due to Infection

Virgin female wild-type and TLR2^−/−^ mice, aged 8–12weeks, were mated with males of the same strain. The vulvas of the female mice were observed at 08:00, and a visible vaginal plug was deemed to indicate day 0.5 of pregnancy [gestational day 0.5 (Gd0.5)]. The female mice were weighed daily, and mice with abdominal distension at Gd12.5 were deemed to be pregnant. The pregnant mice were inoculated intraperitoneally with *T. gondii* tachyzoites (1.0×10^4^/0.4ml RPMI-1640 medium) on Gd12.5. Uninfected pregnant mice from both groups were inoculated intraperitoneally with 0.4ml of RPMI-1640 medium on Gd12.5. A normal birth was defined as “when the weight of the pregnant mouse increased day by day and birth occurred between Gd19.5 and Gd20.5,” and all other pregnancies were considered abnormal. Abnormal pregnancies included premature births and stillbirths.

The definition of premature birth was “when delivery occurred earlier than the scheduled date” (McCarthy et al., 2018), and the definition of stillbirth was “when weight loss was observed in the pregnant mouse for two consecutive days and the pups died *in utero*, detected with laparotomy” (Rahman et al., 2017). The information on the mice used in the experiment and the outcomes of the births is shown in Supplementary Table S1. Samples were collected immediately after birth in cases of normal and premature births. In cases of stillbirth, samples were collected at the time of confirmation of the death of the pups with laparotomy. For the sampling, live animals were euthanized. Blood samples were collected for the quantitative analysis of serum cytokines. Uteri were collected for the quantitative analysis of parasite numbers. Half the placentas were used for histopathological analysis, and the other half were used to quantify the numbers of parasites and for reverse transcription–real-time quantitative PCR (RT–qPCR) analysis. Information on the samples collected in the postpartum period, including at stillbirth and at Gd18.5 [6days postinfection (dpi)], is shown in Supplementary Tables S2 and S3, respectively.

### Quantitative PCR Analysis of Parasite Numbers

The numbers of parasites in the tissues collected from the wild-type and TLR2^−/−^ mice were quantified with a qPCR analysis of *T. gondii* DNA per 50ng of tissue DNA. For DNA preparation, the tissues were lysed with 10 times the weight of the tissue in extraction buffer (0.1M Tris–HCl [pH 9.0], 1% SDS, 0.1M NaCl, and 1mM EDTA) and 100μg/ml of proteinase K (Sigma) and incubated at 50°C. DNA was extracted from the tissues with phenol–chloroform–isoamyl alcohol (25:24:1; Sigma), followed by DNA purification by ethanol. Quantitative PCR was performed with the ABI Prism 7900HT Sequence Detection System (Applied Biosystems, Bedford, MA, United States), with Power SYBR^™^ Green PCR Master Mix (Applied Biosystems), according to the product protocol. The parasite DNA was amplified with primers specific for the *T. gondii B1* gene: forward primer B1-B22 (5'-AACGGGCGAGTAGCACCTGAGGAGA-3') and reverse primer B1-B23 (5'-TGGGTCTACGTCGATGGCATGACAAC-3'; Contini et al., 2005). The parasite numbers were calculated with standard curve interpolation on a plot of the cycle threshold (CT) values against known concentrations of the parasite (serially diluted samples ranging from 10,000 to 0.01 parasites; Tanaka et al., 2013).

### Histopathological Analysis

The placental tissues were fixed in 10% neutral-buffered formalin solution for 1–2weeks at room temperature, dehydrated, and embedded in paraffin, according to the standard method, using a sealed automatic fixation and embedding system (Tissue-Tek VIP 5 Jr., Sakura, Tokyo, Japan). Paraffin blocks were prepared with a paraffin-embedded block preparation system (Tissue-Tek TEC^™^, Sakura). The paraffin blocks were sliced to 3-μm thickness to prepare the tissue sections, which were stained with hematoxylin and eosin (HE). The tissues were observed with an all-in-one fluorescence microscope (BZ-9000; Keyence, Osaka, Japan) and the BZ-II analysis software (Keyence). All sections were analyzed in a blinded manner. The resultant slides were observed by two veterinary pathologists (N.U. and H.F.).

### Immunohistochemistry for Caspase-3 and Image Analysis

Immunohistochemistry for caspase-3 was performed to examine apoptosis in the placentas at Gd18.5. After deparaffinization, antigen was retrieved by microwave oven with citrate buffer (pH 6.0) at 98°C for 15min. In order to inactivate endogenous peroxidase, the sections were immersed in 3% hydrogen peroxide in methanol for 5min. Subsequently, the sections were washed with Tris-buffered saline (TBS) and then blocked with 8% skim milk in TBS at 37°C for 40min. After incubation with the primary antibodies for caspase-3 (Cat# ab13847, Abcam, Cambridge, United Kingdom) at 4°C overnight, the sections were incubated with the DAKO EnVision+ system horseradish peroxidase-labeled polymer anti-rabbit secondary antibody (Dako, Tokyo, Japan) at 37°C for 40min. Immunolabeled antigens were visualized using 0.05% 3,3'-diaminobenzidine plus 0.03% hydrogen peroxide in a Tris-hydrochloric acid buffer and then counterstained with hematoxylin. The severity of apoptosis in the placenta was quantified using an image-processing method. Immunopositive areas were measured using ImageJ software (National Institutes of Health, Bethesda, MD). Briefly, immunopositive pixels were counted in five randomly selected fields in the placenta.

### Real-Time PCR Analysis

To measure mRNA expression, placental tissues were collected from the pregnant mice in the following groups: infected wild-type mice, uninfected wild-type mice, infected TLR2^−/−^ mice, and uninfected TLR2^−/−^ mice. The tissues were crushed with a Bio Masher (Nippi, Tokyo, Japan). The total RNA was extracted with Tri Reagent (Sigma) and then washed with ethanol. cDNA was synthesized from 500ng of total RNA with Prime Script^™^ RT Master Mix (Takara, Kusatsu, Japan), according to the product protocol. Real-time PCR was performed with the ABI Prism 7900HT Sequence Detection System (Applied Biosystems), with Power SYBR^™^ Green PCR Master Mix (Applied Biosystems), according to the product protocol. The relative amounts of mRNA were calculated with the ddCt method, according to the manufacturer’s instructions (User Bulletin no. 2; PerkinElmer, Boston, MA, United States). The primer sequences, designed with the Primer Express software (Applied Biosystems), were: β-actin sense primer 5'-GCTCTGGCTCCTAGCACCAT-3', β*-*actin antisense primer 5'-GCCACCGATCCACACAGAGT-3'; glyceraldehyde 3-phosphate dehydrogenase (GAPDH) sense primer 5'-TGTGTCCGTCGTGGATCTGA-3', GAPDH antisense primer 5'-CCTGCTTCACCACCTTCTTGAT-3'; IL-12p40 sense primer 5'-CGCAGCAAAGCAAGATGTGT-3', IL-12p40 antisense primer 5'-TGGCAAACCAGGAGATGGTT-3'; IL-6 sense primer 5'-TTCCATCCAGTTGCCTTCTTG-3', IL-6 antisense primer 5'-GAAGGCCGTGGTTGTCACC- 3'; TNF-α sense primer 5'-GGCAGGTCTACTTTGGAGTCATTGC-3', TNF-α antisense primer 5'-ACATTCGAGGCTCCAGTGAA-3'; IL-4 sense primer 5'-CACGGATGCGACAAAAATCA-3', IL-4 antisense primer 5'-CTCGTTCAAAATGCCGATGA-3'; and IL-10 sense primer 5'-AAGGGTTACTTGGGTTGC-3', IL-10 antisense primer 5'-AAGGAGTTGTTTCCGTTA-3'. Foxp3 sense primer 5'-GAGAAAGCGGATACCAAA-3', antisense primer 5'-TGTGAGGACTACCGAGCC-3'. The β-actin mRNA was used as the internal control gene after comparison with GAPDH mRNA using RefFinder (Xie et al., 2012). Gene-specific expression was normalized to the expression of the mouse β-actin mRNA.

### Analyses of Cytokine and Prostaglandin E2

Blood samples were collected from the mice, and the serum concentrations of IFN-γ and IL4 were measured with enzyme-linked immunosorbent assays (ELISAs; BD Bioscience, San Jose, CA, United States), according to the manufacturers’ protocol. The concentration of prostaglandin E2 in sera was also tested with an enzyme immunoassay kit (Cayman Chemical Co., MI, United States).

### Statistical Analysis

The analysis software used was GraphPad Prism software (GraphPad Software Inc., La Jolla, CA, United States). Data are presented as means ± standard deviations (SD). Statistical analyses were performed with Fisher’s exact test, one-way analysis of variance (ANOVA), two-way ANOVA, F-test, Student’s *t* test, or the Mann–Whitney *U* test. The levels of statistical significance are shown with asterisks and are defined in each figure legend, together with the name of the statistical test used. *p* values of <0.05 were considered statistically significant.

## Result

### Abnormal Pregnancy Caused by *T. gondii* Infection

**Figure 1:**
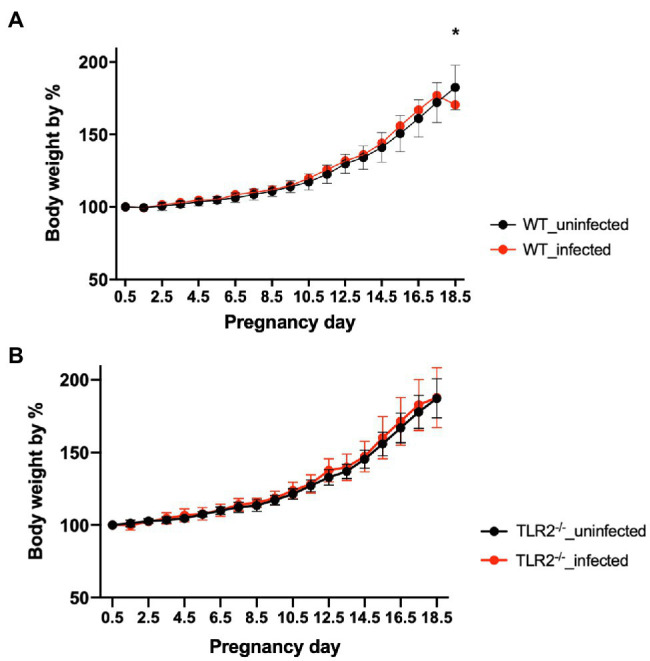
Bodyweight changes in uninfected mice and mice infected with *T. gondii*. Values presented are the means ± standard deviations of each group. **(A)** Bodyweight changes in uninfected wild-type (WT) and *T. gondii*-infected WT mice. **(B)** Bodyweight changes in uninfected TLR2^−/−^ and *T. gondii*-infected TLR2^−/−^ mice. The statistical analysis was performed with two-way ANOVA and Bonferroni’s multiple-comparison test. ^*^p<0.05.

**Figure 2:**
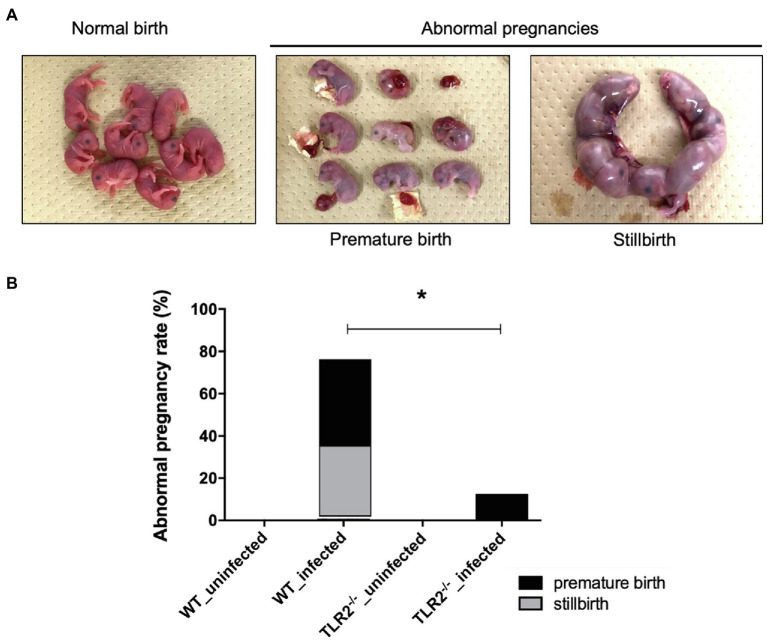
Abnormal pregnancy rates in mice uninfected and infected with *T. gondii*. **(A)** Pups from normal births and dead pups from abnormal pregnancies, such as premature births and stillbirths. Representative photographs are shown. **(B)** Abnormal pregnancy rates in uninfected and infected mice. Abnormal pregnancy rates in each group are shown in a bar graph (uninfected wild-type [WT] mice, 0/12, 0%; infected WT mice, 9/12, 75%; uninfected TLR2^−/−^ mice, 0/5, 0%; infected TLR2^−/−^ mice, 1/8, 12.5%). Statistical analysis was performed with Fisher’s exact test. ^*^p<0.05.

Although there was no significant difference in the bodyweight gain between the uninfected TLR2^−/−^ mice and the infected TLR2^−/−^ mice, the mean bodyweight of the infected wild-type mice at Gd18.5 was lower than that of the uninfected wild-type mice (Figure 1). Among the mice infected with *T. gondii*, some animals had abnormal pregnancies, including premature births and stillbirths (Figure 2A). The abnormal pregnancy rate was 75% (9/12) in the infected wild-type mice and 12.5% (1/8) in the infected TLR2^−/−^ mice. This result indicates significantly higher abnormal pregnancy rates in the infected wild-type mice, whereas both types of uninfected mice also had normal pregnancies and births (Figure 2B, Supplementary Table S1).

### Impact of *T. gondii* Infection on Pups and Placentas

**Figure 3:**
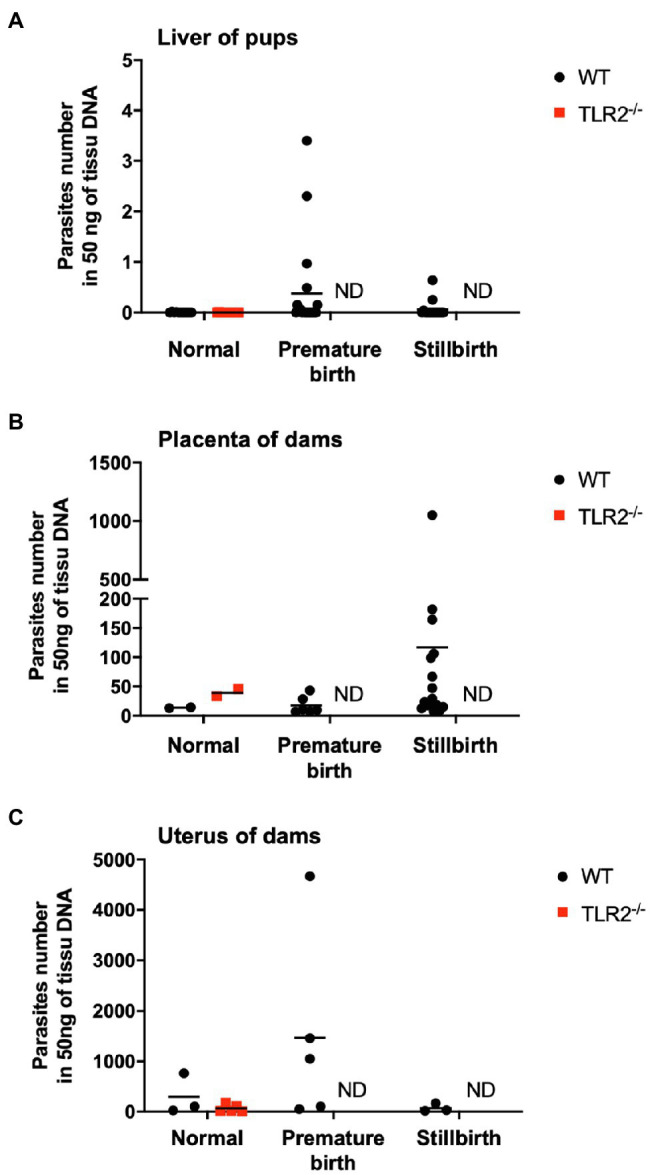
Quantification of the numbers of parasites in the livers of pups and the placentas and uteri of dams. Numbers of parasites in the livers of pups **(A)**, placentas of dams **(B)**, and uteri of dams **(C)** from infected wild-type (WT) and TLR2^−/−^ mice were analyzed with quantitative PCR. Each point on the graph represents one tissue, and the horizontal lines indicate the mean values for each group. **(A)** Normal birth: WT (*n*=11), TLR2^−/−^ (*n*=14); premature birth: WT (*n*=20), TLR2^−/−^ (*n*=0); stillbirth: WT (*n*=15), TLR2^−/−^ (*n*=0). **(B)** Normal: WT (*n*=2), TLR2^−/−^ (*n*=2); premature birth: WT (*n*=6), TLR2^−/−^ (*n*=0); stillbirth: WT (*n*=16), TLR2^−/−^ (*n*=0). **(C)** Normal: WT (*n*=3), TLR2^−/−^ (*n*=5); premature birth: WT (*n*=5), TLR2^−/−^ (*n*=0); stillbirth: WT (*n*=3), TLR2^−/−^ (*n*=0). Statistical analysis of normal birth samples was performed with the Mann–Whitney *U* test or *t* test, but no differences were detected. ND: not done because no sample was available.

**Figure 4:**
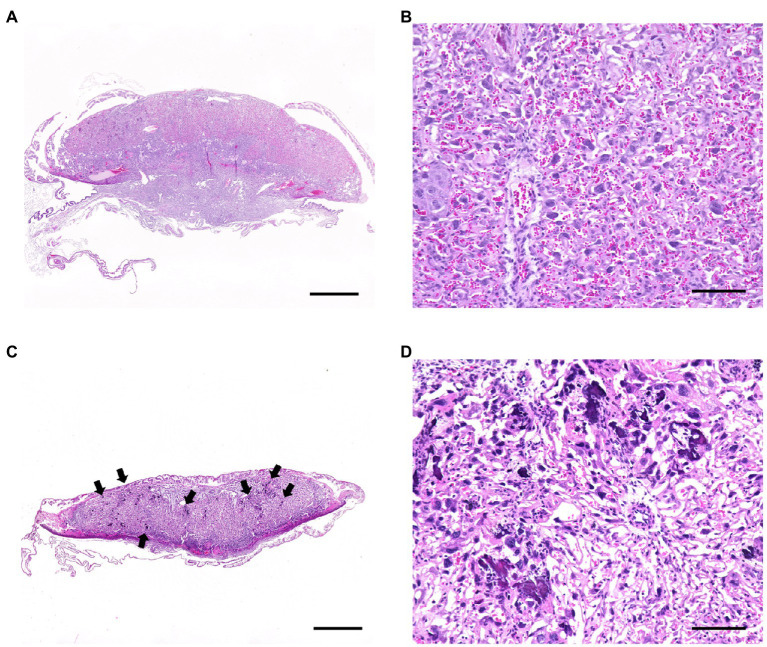
Histological changes in the placentas of *T. gondii*-infected wild-type mice with abnormal pregnancies. Tissue sections from the placentas of infected wild-type mice with abnormal pregnancies showed multiple calcifications in 19 of 25 samples. Representative photographs are shown. **(A)** Normal placenta on day 18.5 of pregnancy (Gd18.5). Bar=1.0mm. **(B)** High magnification of the labyrinth of the normal placenta. A large number of mature erythrocytes were observed in the vascular spaces of the labyrinth. Bar=100μm. **(C)** Placenta of a stillbirth mouse at Gd19.5. Bar=1.0mm. Multifocal calcification (arrows) were observed at the labyrinth of the placenta. A few of mature erythrocytes were observed in the vascular spaces of the labyrinth. **(D)** High magnification of the labyrinth. Bar=100μm.

We examined the pups and placentas in the postpartum period, including stillbirths, because fetal and placental dysfunction are common causes of pregnancy failure. The number of parasites in the livers of the pups from the infected wild-type and infected TLR2^−/−^ mice was quantified (Figure 3A). A small number of *T. gondii* (<4.0 parasites) were detected in the infected wild-type mice (2/48), but not in the infected TLR2^−/−^ mice (0/14), indicating little vertical transmission of *T. gondii* in either type of mouse (Figure 3A). In the wild-type mice, relatively higher numbers of parasites were detected in the placentas associated with stillbirths and the uteri associated with premature births, but they did not differ significantly from the samples associated with normal pregnancies (Figures 3B,C). A histological comparison of the brains and livers of the wild-type and TLR2^−/−^ pups in the uninfected and infected groups showed no histological changes attributable to infection (Supplementary Figure S1). The placentas of the infected wild-type mice with abnormal pregnancies were analyzed histologically, and multiple calcifications were observed in seven of the eight mice (Figure 4 and Supplementary Figure S2). We confirmed one case of premature birth in the infected TLR2^−/−^ mice, but we could not collect the placenta.

### Immunological Response to *T. gondii* Infection

**Figure 5:**
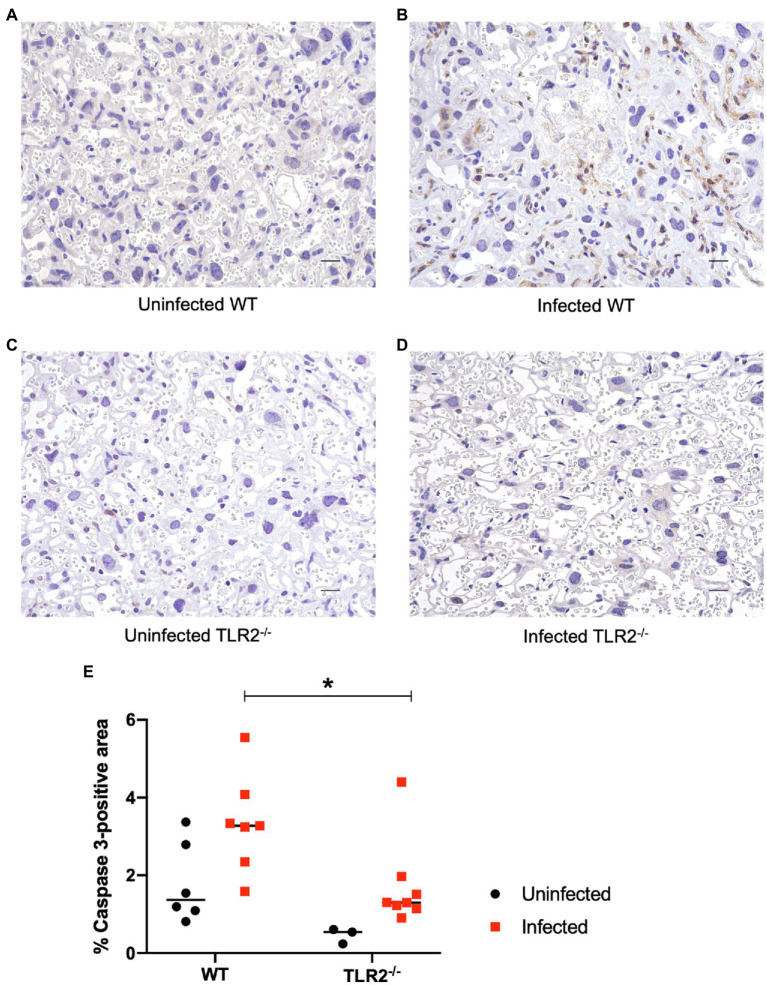
Immunohistochemistry and image analysis for caspase-3 in the placenta on day 18.5 of pregnancy. Trophoblasts and endothelial cells were positive for caspase-3 in the placenta of the infected wild-type mice. Representative photographs are shown. **(A)** The placenta of an uninfected wild-type mouse. **(B)** The placenta of an infected wild-type mouse. **(C)** The placenta of an infected TLR2^−/−^ mouse. **(D)** The placenta of an infected TLR2^−/−^ mouse. Bar=20μm. Trophoblasts and endothelial cells were positive for caspase-3 in an infected wild-type mice. **(E)** Image analysis of immunihistochemistry for caspase-3. Positive areas were compared between WT and TLR2^−/−^ mice. Horizontal bars represent the mean value. Data were analyzed with two-way ANOVA followed by the Tukey–Kramer multiple-comparison test. ^*^p<0.05.

To confirm the inflammatory response in terms of pathological changes in the placenta, histological and molecular analyses were performed at Gd18.5 (6 dpi). Although higher parasite numbers were detected in the placentas and uteri of some TLR2^−/−^ mice, there was no significant difference in the parasite numbers between the wild-type and TLR2^−/−^ mice (Supplementary Figure S3). A histological comparison of the placentas of the uninfected and infected groups showed no histological changes attributable to infection in either the wild-type or TLR2^−/−^ mice (Supplementary Figure S3). Immunohistochemistry, however, revealed that trophoblasts and endothelial cells of the placenta were strongly positive for caspase-3 in the infected wild-type mice (Figure 5B), compared with the uninfected wild-type and TLR2^−/−^ mice (Figures 5A,C) and the infected TLR2^−/−^ mice (Figure 5D). Image analysis revealed that caspase-3-positive area was significantly increased in the infected wild-type mice than the infected TLR2^−/−^ mice (Figure 5E).

**Figure 6:**
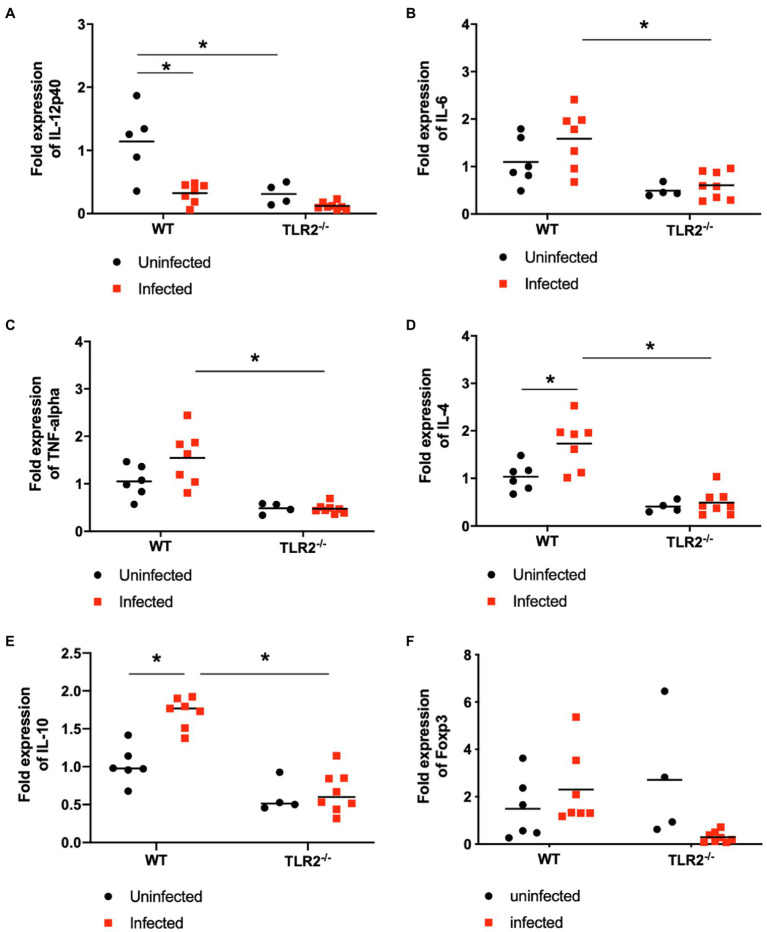
Relative mRNA expression of cytokines in placenta on day 18.5 of pregnancy. Levels of IL-12p40 (A), IL-6 (B), TNF-a (C) IL-4 (D), IL-10 (E) and Foxp3 (F) mRNAs in the placentas of each group were measured with RT PCR. Using synthesized cDNA, the mRNA expression levels of cytokines relative to those in uninfected wild-type (WT) mice were quantified with RT qPCR. Because embryos and placentas collected from one mouse were pooled into one sample, each symbol indicates the data from a single mouse. Uninfected WT mice (*n*=6), infected WT mice (*n*=7), uninfected TLR2^−/−^ mice (*n*=4), infected TLR2^−/−^ mice (*n*=8). The horizontal lines show the mean values for each group. Data were analyzed with two-way ANOVA followed by the Tukey–Kramer multiple-comparison test. ^*^p<0.05.

The levels of IL-12p40, IL-6, TNF-α, IL-4, and IL-10 mRNAs in the placenta were measured with qPCR (Figure 6). In the wild-type mice, *T. gondii* infection increased IL-4 and IL-10 mRNA expression and reduced IL-12p40 mRNA expression compared with that in the uninfected mice. The level of IL-12p40 mRNA in the uninfected TLR2^−/−^ mice was significantly lower than that in the uninfected wild-type mice and was similar to that in the infected TLR2^−/−^ mice. The levels of IL-6, TNF-α, IL-4, and IL-10 mRNAs were significantly lower in the infected TLR2^−/−^ mice than in the infected wild-type mice. However, statistically significant difference was not seen in Foxp3 expression among the groups.

**Figure 7:**
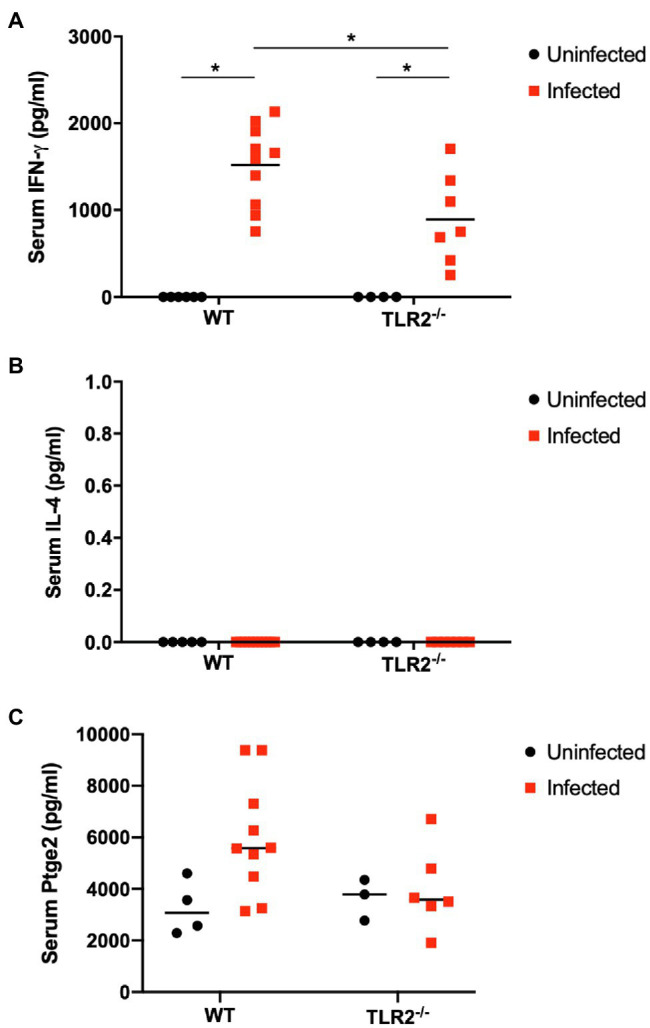
Serum levels of IFN-γ (A), IL4 (B) and prostaglandin E2 (C) on day 18.5 of pregnancy. Serum levels of IFN-γ, IL4 and prostaglandin E2 in each group were measured. Each point on the graph represents a single mouse, and the horizontal lines indicate the mean values for each group. Uninfected WT mice (*n*=6), infected WT mice (*n*=10), uninfected TLR2^−/−^ mice (*n*=4), infected TLR2^−/−^ mice (*n*=7). Data were analyzed with two-way ANOVA followed by the Tukey–Kramer multiple-comparison test. ^*^p<0.05.

The IFN-γ and IL-4 levels in the mouse sera were measured with ELISAs as indicators of their systemic immune status. Serum IFN-γ levels were increased significantly by *T. gondii* infection in both the wild-type mice and TLR2^−/−^ mice, but the IFN-γ levels were significantly lower in the infected TLR2^−/−^ mice than in the infected wild-type mice (Figure 7). Serum IL4 was not detectable in this study. Additionally, serum prostaglandin E_2_ was measured because TLR2 is important to trigger prostaglandin E_2_ production (Umeda et al., 2017) and prostaglandin E_2_ can favor the parasite proliferation in trophoblast cells (Barbosa et al., 2014). As shown in Figure 7C, *T. gondii* infection showed a tendency to increase serum prostaglandin E_2_ in wild-type mice, not in TLR2^−/−^ mice, while there was no statistically significant difference among the experimental groups.

## Discussion

The maternal immune system plays two major roles against pathogen infection during pregnancy: It maintains the pregnancy and combats the pathogen (Robinson and Klein, 2012; Rowe et al., 2013). However, immunomodulation in the placenta is strongly affected by pathogens, resulting in a high pathogen burden at the fetal–maternal interface, which causes pregnancy complications such as abortion and stillbirth (Gibbs, 2002; Goldenberg et al., 2010; Robbins and Bakardjiev, 2012). The infection of pregnant animals with *T. gondii* can cause embryonic death, fetal resorption, fetal death, abortion, and congenital transmission (Dubey, 2009). Furthermore, infection with *T. gondii* during early pregnancy has more severe consequences (e.g., fetal resorption, reduced offspring survival rates, and increased parasite transmission rates) than infection during late pregnancy (Shirahata et al., 1992; Montoya and Liesenfeld, 2004; Wang et al., 2011). Although the effects of *T. gondii* infection during late pregnancy are poorly understood, changes in the immune response and interactions with PRRs may be associated with pregnancy complications. Because TLR2 is expressed in trophoblast cells in late gestation (Abrahams et al., 2004; Rindsjö et al., 2007), in the present study, we examined the roles of TLR2 in late pregnancy after *T. gondii* infection.

The rate of bodyweight gain at Gd18.5 was significantly lower in the infected wild-type mice than in the uninfected wild-type mice (Figure 1A), suggesting that some mice lost bodyweight through premature birth or stillbirth triggered by *T. gondii* infection. In contrast, the infected TLR2^−/−^ mice showed no reduction in bodyweight gain (Figure 1B). In this study, the pregnant mice were inoculated intraperitoneally with *T. gondii* tachyzoites (1.0×10^4^/0.4ml RPMI-1640 medium) on Gd12.5. So, Gd 18.5 is 6days post-infection. Generally, clinical symptoms in *T. gondii*-infected C57BL/6 mice are seen after 5–7days postinfection. Therefore, despite the fact that C57BL/6 is known to be highly susceptible to *T. gondii* infection, there was almost no difference between groups on body weight. Moreover, the TLR2^−/−^ mice had significantly fewer abnormal pregnancies in response to *T. gondii* infection than the infected wild-type mice (Figure 2B, Supplementary Table S1). These results suggest that TLR2 is involved in disease exacerbation during *T. gondii* infection in late pregnancy.

The damage to the placenta and pups caused by *T. gondii* infection is a factor causing pregnancy failure in mice (Coutinho et al., 2012; Zhang et al., 2015; Vargas-Villavicencio et al., 2016). Moreover, infection of the placenta and pups has been detected in *Calomys callosus* (a South American rodent of the family Cricetidae, often used in experiments involving acute *T. gondii* infection; Ferro et al., 2002). In the present study, few parasites were detected in the pups from the infected dams, suggesting the low vertical transmission of *T. gondii* under our experimental conditions. Previous study also showed that placental infection of *T. gondii* was seen in C57BL/6 mice infected with 30 cysts of *T. gondii* (type II strain) at Gd11.0, while no vertical transmission was observed (Shiono et al., 2007). A histopathological analysis of sheep neonates experimentally infected with *T. gondii* during pregnancy showed leukomalacia, glial cell infiltration in the brain, and the necrosis of multiple tissues throughout the whole body (Castaño et al., 2016). However, our histopathological analysis of the brains and livers of mouse pups indicated that no histological changes were caused by *T. gondii* infection during late pregnancy. However, the *T. gondii B1* gene was detected in placentas and uteri of the infected dams. These results suggest that *T. gondii* infection does not cause pathological changes in the pups because the placenta is a barrier to its vertical transmission when infection occurs during late pregnancy (Robbins and Bakardjiev, 2012).

Human, rats and mice have hemochorial type of placentas, characterized by a limited cellular barrier without the maternal tissue layer (Furukawa et al., 2014). Cellular barrier is composed of one to three layers of cytotrophoblasts and syncytiotrophoblasts (Soares et al., 2018). An *in vitro* analysis of human placenta suggested that the placenta is a barrier to *T. gondii* infection and indicated the particular importance of syncytiotrophoblasts (Robbins et al., 2012). Although there are some differences in the structures of human and mouse placentas, syncytiotrophoblasts may also act as a barrier in mice (Maltepe et al., 2010). A histopathological analysis of the placentas from the infected mice with abnormal pregnancies showed multiple calcifications in seven of the eight mice (Figures 4C,D). Because calcinosis occurs when calcium salts are deposited at the site of cell or tissue death (dystrophic calcification), an inflammatory response occurs in the placenta, resulting in cell death. The results of placental infection with *T. gondii* and calcification suggest that *T. gondii* infection during late pregnancy is involved in the deterioration of placental function. The death of pups in a dam (stillbirth) is presumed to be caused by a lack of oxygen and the nutrients required by the pups, arising from the deterioration of the placenta. No histological changes were observed in the pups because histological lesions rarely appear in cases of rapid death due to lack of oxygen. Because multiple calcifications were not seen in the placentas of the TLR2^−/−^ mice, TLR2 signaling during late pregnancy may be involved in this placental damage.

To investigate the immune changes caused by *T. gondii* infection during pregnancy, we analyzed the parasite burden and the immune response at Gd18.5 (6 dpi). Unexpectedly, there were no significant differences in the parasite numbers in the placentas and uteri of the wild-type and TLR2^−/−^ mice (Supplementary Figure S3). We found higher parasite numbers in the placentas and uteri of some TLR2^−/−^ mice, possibly because TLR2 plays an important role in the elimination of the parasite by inducing a Th1 immune response. In addition, prostaglandin E_2_ induced by *T. gondii* infection might support the parasite proliferation in trophoblast of wild-type mice, not TLR2^−/−^ mice (Figure 7C; Barbosa et al., 2014). Furthermore, no histopathological changes were seen in the placentas of either group of animals (Supplementary Figure S4). However, some differences in cytokine mRNA expression were detected in the placentas (Figure 6). *T. gondii* infection downregulated the IL-12p40 mRNA expression and upregulated the mRNA expression of IL-4 and IL-10 in the placenta at Gd18.5 (6 dpi). These results contradict a report that *T. gondii* type II strains, including PLK, stimulate IL-12 production in an MYD88-dependent manner in nonpregnant animals (Robben et al., 2004; Kim et al., 2006). *In vitro* studies with the human placental cell line BeWo showed that the expression of the proinflammatory cytokines IL-6 and TNF-α is downregulated by high concentrations of IFN-γ (Barbosa et al., 2015). In the present study, *T. gondii* infection significantly increased the serum levels of IFN-γ, a known proinflammatory cytokine (Figure 7). Therefore, in the placenta, high levels of IFN-γ may downregulate the expression of the Th1 cytokine IL-12p40, resulting in the upregulation of the Th2 cytokines IL-4 and IL-10, to maintain the Th1/Th2 balance.

Macrophages at the maternal–fetal interface characterized by alternative activation (M2 polarization) exhibit immunosuppressive functions for the maintenance of pregnancy. However, *T. gondii* infection changes the bias of M2 decidual macrophages toward M1, resulting in the immunosuppressive microenvironment at the maternal–fetal interface and the adverse pregnancy outcomes (Liu et al., 2018). IL-4 plays an important role in the maintenance of pregnancy by reducing the inflammatory response. IL-4 induces M2 macrophages, which are responsible for the production of the growth factors and anti-inflammatory cytokines that promote wound healing and tissue remodeling, and improves the metabolism and endocrine signaling in tissues (Murray and Wynn, 2011). Like IL-4, IL-10 is a cytokine involved in the Th2 response. In nonpregnant mice infected with *T. gondii*, IL-4 deficiency increased mortality, but reduced the brain tissue cyst burden (Roberts et al., 1996; Alexander et al., 1998). In contrast, significant mortality occurred immediately postpartum in wild-type dams, whereas all IL-4-deficient dams survived (Alexander et al., 1998). These results and our present data suggest a role for IL4 in pregnancy-induced immunomodulation and the associated increased susceptibility to *T. gondii* infection. According to a report by Gazzinelli et al. (1996), nonpregnant mice lacking IL-10 produced excessive amounts of IL-12, IFN-γ, and TNF-α in response to *T. gondii* infection, resulting in a lethal immune response, with severe cellular infiltration and necrosis in the liver (Gazzinelli et al., 1996). In contrast to IL-4, IL-10 reduces the apoptosis of decidual regulatory T cells (Treg cells), improving adverse pregnancy outcomes after *T. gondii* infection (Lao et al., 2015). The higher levels of IL-10 mRNA expression seen in the placentas of the infected wild-type dams in the present study require further research. Together, the changes in cytokine expression in the placenta after *T. gondii* infection during pregnancy may affect the parasite burden and calcinosis in the placenta. Moreover, TLR2 is involved in this disease outcome because no changes in the expression levels of IL-12p40, IL-6, TNF-α, or IL-4 were observed in the TLR2^−/−^ mice during *T. gondii* infection (Figure 6).

Several kinds of immune cells should be considered for maintenance of pregnancy. Worse pregnancy outcomes observed in mice were associated with higher TNF-α and IL-6 at the maternal–fetal interface, with lower Foxp3 expression (Sousa et al., 2021). Because Foxp3 is an essential molecular marker of regulatory T cell (Treg) development (Kim and Rudensky, 2006), Treg plays an important role on maintenance of pregnancy. *T. gondii* infection significantly downregulates the number of whole decidual Treg cell population by upregulating the suppressive function of programmed cell death protein 1 (PD-1)^+^ Treg cell population (Zhang H et al., 2019) and apoptosis (Qiu et al., 2018), resulting in adverse pregnancy outcomes. On the other hand, Treg cell population and transforming growth factor-beta (TGF-β) level contribute to pregnancy outcomes (Xu et al., 2017; Zhao et al., 2017). Thus, immune inhibitory activity is one of the important factors. However, mRNA expression of Foxp3 in placenta on day 18.5 of pregnancy did not show significant difference among the experimental groups in our study (Figure 6F).

Immune inhibitory molecules such as T cell immunoglobulin and mucin domain-containing protein 3 (Tim-3) and leukocyte immunoglobulin-like receptor subfamily B member 4 (LILRB_4_) contribute on the pregnancy outcome. The Tim-3 is discovered to be expressed on some decidual immune cells and participates in the maintenance of maternal–fetal tolerance. Dysregulation of Tim-3 expression on decidual NK (dNK) cells and decidual macrophages was observed in several cases of pregnancy complications. Tim-3^−/−^ pregnant mice displayed more worse pregnancy outcomes with *T. gondii* infection (Zhang D et al., 2019; Li et al., 2021). LILRB_4_ expressed by macrophages and dendritic cells plays an important immune-regulatory role at the maternal–fetal interface. *T. gondii* infection strengthens M1 activation functions and weakened M2 tolerance functions by downregulation of LILRB4 on decidual macrophages (Li et al., 2017). Moreover, LILRB4 expression on uterine dendritic cells subsets from mid-gestation is obviously downregulated after *T. gondii* infection (Zhan et al., 2018). Contribution of Tim-3 and LILRB_4_ should be clarified in our experimental models for future study.

We consider that the calcification of the placenta observed in the infected wild-type mice was due to cell death *via* a caspase-3 pathway caused by increased levels of IFN-γ in the blood. IFN-γ is a well-known cytokine that is important for the elimination of *T. gondii* in nonpregnant animals (Sturge and Yarovinsky, 2014). However, an *in vitro* study reported that IFN-γ did not prevent the growth of *T. gondii* in BeWo cells (Barbosa et al., 2015), suggesting that the primary role of IFN-γ in the placenta may be other than the inhibition of parasite growth. One mechanism of pregnancy failure involving TLR2 involves the apoptosis of trophoblast cells. An *in vitro* study using human placentas showed that the administration of peptidoglycan, a ligand of TLR2, caused the apoptosis of trophoblast cells (Abrahams et al., 2004), and an *in vivo* study of pregnant mice showed that the intrauterine administration of peptidoglycan induced preterm delivery, mainly characterized by apoptosis (Ilievski et al., 2007). IFN-γ is associated with the apoptosis of trophoblasts *via* a caspase-3- and caspase-8-dependent pathway (Zhang et al., 2015). Senegas et al. reported that the IFN-γ-dependent apoptosis of placental cells is involved in embryo resorption in *T. gondii*-infected mice during early pregnancy (Senegas et al., 2009). In the present study of late pregnancy, the apoptosis and dystrophic calcification of trophoblast cells in the infected wild-type mice may have been induced by the production of IFN-γ *via* the activation of TLR2. However, the production of IFN-γ was lower in the infected TLR2^−/−^ mice than in the infected wild-type mice, resulting in less induction of trophoblast apoptosis and fewer dams with abnormal pregnancies.

Cell death in the placenta reduces its ability to act as a physical barrier to infection by *T. gondii* and may promote the transmission of the parasite to the fetus. An *in vitro* study using human placenta by Robbins et al. (2012) reported that *T. gondii* was abundant at sites of syncytiotrophoblast disruption, suggesting that placental damage and the vertical transmission of *T. gondii* are related. In the present study, fewer parasites were detected in the livers of pups from infected TLR2^−/−^ mice than in the pups from the infected wild-type mice (Figure 3A). Our results suggest that the infected TLR2^−/−^ mice were less likely to experience apoptosis of the placenta caused by IFN-γ and that the physical barrier function of the placenta against *T. gondii* was not compromised. Therefore, TLR2^−/−^ mice may be more resistant to the vertical transmission of *T. gondii*.

Our results indicate that TLR2 signaling makes a major contribution to abnormal pregnancies after *T. gondii* infection. Notably, TLR2 signaling is important because glycosylphosphatidylinositol is a parasite ligand of TLR2 and TLR4 (Debierre-Grockiego et al., 2007). Therefore, the regulation of TLR2 signaling may be a potential target in controlling congenital toxoplasmosis.

## Data Availability Statement

The original contributions presented in the study are included in the article/Supplementary Material, further inquiries can be directed to the corresponding author.

## Ethics Statement

The animal study was reviewed and approved by the Committee on the Ethics of Animal Experiments at Obihiro University of Agriculture and Veterinary Medicine, Obihiro, Japan (permit numbers 18-50, 19-56, 20-27).

## Author Contributions

RI was involved in data curation, formal analysis, investigation, and writing—original draft. NU contributed to investigation and writing—review and editing. AA performed investigation. HF done data curation, investigation, methodology, and writing—review and editing. YN was involved in data curation, formal analysis, investigation, writing—review and editing, conceptualization, project administration, funding acquisition, and supervision. All authors contributed to the article and approved the submitted version.

## Funding

This study was supported in part by the Research Program on Emerging and Re-emerging Infectious Diseases [20fk0108137h (YN)] from the Agency for Medical Research and Development (AMED), KAKENHI Grants from the Japan Society for the Promotion of Science [20KK0152, 20K21359, 21H02353 (YN)].

## Conflict of Interest

The authors declare that the research was conducted in the absence of any commercial or financial relationships that could be construed as a potential conflict of interest.

## Publisher’s Note

All claims expressed in this article are solely those of the authors and do not necessarily represent those of their affiliated organizations, or those of the publisher, the editors and the reviewers. Any product that may be evaluated in this article, or claim that may be made by its manufacturer, is not guaranteed or endorsed by the publisher.
