# Supplementary material for: Toll-Like Receptor 2 is Involved in Abnormal Pregnancy in Mice Infected with Toxoplasma gondii During Late Pregnancy
Source: Front Microbiol. 2021 Oct 5;12:741104. doi: 10.3389/fmicb.2021.741104 (PMC8524087; doi:10.3389/fmicb.2021.741104)
Supplement: Supplementary file 1 [file Data_Sheet_1.docx]

**Supplemental information file**

**Table S1**. **Abnormal pregnancy rates in mice uninfected and infected with *T. gondii*.**

| Mouse group | Number of dam mice | Normal births | Abnormal pregnancies | Abnormal pregnancy rate (%) | Details of abnormal pregnancy |
| --- | --- | --- | --- | --- | --- |
| Infected wild-type | 12 | 3 | 9 | 75.0 | Premature birth (n = 5) Stillbirth (n = 4) |
| Uninfected wild-type | 12 | 12 | 0 | 0.0 |  |
| Infected TLR2^−/−^ | 8 | 7 | 1 | 12.5 | Premature birth (n = 1) |
| Uninfected TLR2^−/−^ | 5 | 5 | 0 | 0.0 |  |

**Table S2**. **Mice and experimental design in the postpartum period, including stillbirths.**

| Mouse group | Number of mice† | Liver of pups for analysis of the parasite number | Placentae for analysis of the parasite number | Uteruses for analysis of the parasite number | Histopathological analysis of pups | Histopathological analysis of placentae |
| --- | --- | --- | --- | --- | --- | --- |
| infected wild-type | 12 | 7^1)^, 2^2)^, 5^3)^, 4^4)^, 4^5)^, 5^6)^, 4^7)^, 4^8)^, 2^9)^, 4^10)^, 4^11)^,3^12)^ | 1^2)^, 4^6)^, 4^7)^, 3^8)^, 2^9)^, 4^10)^, 3^11)^,2^12)^ | 1^1)^, 1^2)^, 1^3)^, 1^4)^, 1^5)^, 1^7)^, 1^8)^, 1^9)^, 1^10)^, 1^11)^,1^12)^ | 1^1)^, 2^2)^, 3^4)^, 4^5)^, 4^6)^, 4^7)^, 4^8)^, 3^9)^, 4^10)^, 2^11)^,3^12)^ | 4^1)^, 4^6)^, 4^7)^, 4^8)^, 3^9)^, 4^10)^, 2^11)^, 2^12)^ |
| Uninfected wild-type | 12 | No data | No data | No data | 4^1)^, 3^2)^, 4^3)^, 4^4)^, 3^5)^, 4^6)^, 4^7)^, 3^8)^, 3^9)^, 4^10)^, 4^11)^,3^12)^ | No data |
| infected TLR2^-/-^ | 8 | 3^1)^, 4^2)^, 1^3)^, 4^4)^, 2^5)^ | 2^4)^ | 1^1)^, 1^2)^, 1^3)^, 1^4)^, 1^5)^ | 3^1)^, 4^2)^, 3^4)^, 2^5)^ | No data |
| uninfected TLR2^-/-^ | 5 | No data | No data | No data | 2^1)^, 2^2)^, 1^3)^, 1^4)^, 1^5)^ | No data |

Superscript numbers denote the mouse identification number per experimental group.

† Denotes the number of mice housed with males overnight, then observed for a vaginal plug. Mice with abdominal distension on Gd12.5 were deemed to be pregnant.

**Table S3**. **Mice and experimental design on day 18.5 of pregnancy (6 days postinfection).**

| Mouse group | Number of mice† | Placentae for analysis of the parasite number | Uteruses for analysis of the parasite number | Histopathological analysis of placentae | mRNA expression analysis of placentae | Cytokine levels analysis of serum |
| --- | --- | --- | --- | --- | --- | --- |
| infected wild-type | 10 | 2^2)^, 4^3)^, 4^4)^, 1^5)^, 5^7)^, 2^8)^, 1^9)^, 1^10)^ | 1^1)^, 1^2)^, 1^3)^, 1^4)^, 1^5)^, 1^6)^, 1^7)^, 1^8)^, 1^9)^, 1^10)^ | 5^3)^, 4^4)^, 1^6)^, 3^7)^, 3^8)^, 4^9)^, 2^10)^ | 1^3)^, 1^4)^, 1^5)^, 1^7)^, 1^8)^, 1^9)^, 1^10)^ | 1^1)^, 1^2)^, 1^3)^, 1^4)^, 1^5)^, 1^6)^, 1^7)^, 1^8)^, 1^9)^, 1^10)^ |
| Uninfected wild-type | 6 | No data | No data | 2^1)^, 3^2)^, 2^3)^, 3^4)^, 3^5)^, 3^6)^ | 1^1)^, 1^2)^, 1^3)^, 1^4)^, 1^5)^, 1^6)^ | 1^1)^, 1^2)^, 1^3)^, 1^4)^, 1^5)^, 1^6)^ |
| infected TLR2^-/-^ | 8 | 3^1)^, 3^2)^, 4^3)^, 3^4)^, 4^5)^, 7^6)^, 6^7)^, 3^8)^ | 1^2)^, 1^3)^, 1^4)^, 1^5)^, 1^6)^, 1^7)^, 1^8)^ | 3^1)^, 4^2)^, 4^3)^, 5^4)^, 3^5)^, 3^6)^, 3^7)^, 2^8)^ | 1^1)^, 1^2)^, 1^3)^, 1^4)^, 1^5)^, 1^6)^, 1^7)^, 1^8)^ | 1^1)^, 1^2)^, 1^4)^, 1^5)^, 1^6)^, 1^7)^, 1^8)^ |
| uninfected TLR2^-/-^ | 4 | No data | No data | 3^1)^, 4^2)^, 4^3)^, 5^4)^ | 1^1)^, 1^2)^, 1^3)^, 1^4)^ | 1^1)^, 1^2)^, 1^3)^, 1^4)^ |

Superscript numbers denote the mouse identification number per experimental group.

† Denotes the number of mice housed with males overnight, then observed for a vaginal plug. Mice with abdominal distension on Gd12.5 were deemed to be pregnant.

**Figure S1.** **Histopathological observations of pups in the postpartum period.** The pups’ brains and livers were used to make tissue sections and the structures were observed. When 34 samples from pups of infected wild-type (WT) dams were compared with 22 samples from pups of uninfected WT dams, postmortem brain autolysis was observed in four samples of pups from an infected WT dam, but no other differences were observed. When 12 samples from pups of infected TLR2^−/−^ dams were compared with seven samples from pups of uninfected TLR2^−/−-^ dams, no differences were found. There were no morphological differences among the samples. Representative photographs are shown.


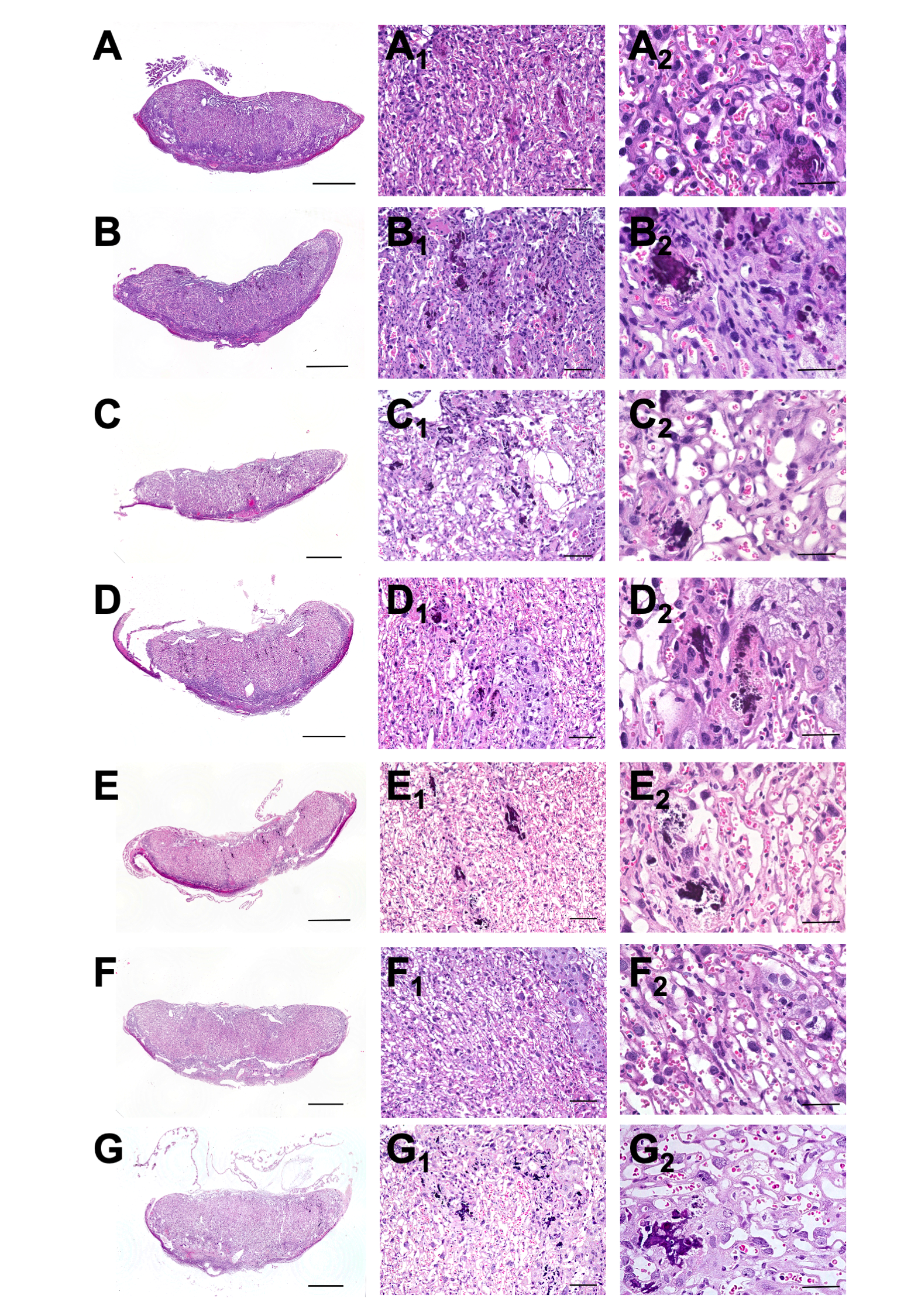


**Figure S2. Histological changes in the placentas of *T. gondii*-infected wild-type mice with abnormal pregnancies.** (A) Placenta of a premature mouse #4^1)^ at Gd18.5. (B) Placenta of a stillbirth mouse #4^6)^ at Gd20.5. (C) Placenta of a stillbirth mouse #4^8)^ at Gd20.5. (D) Placenta of a premature mouse #3^9)^ at Gd18.5. (E) Placenta of a stillbirth mouse #4^10)^ at Gd20.5. (F) Placenta of a premature mouse #2^11)^ at Gd18.5. (G) Placenta of a premature mouse #2^12)^ at Gd18.5. Multifocal calcification at the labyrinth were observed in (A), (B), (C), (D), (E), and (G) without (F). Bar= 1.0 mm (A_1_)-(G_1_) Labyrinth of the placenta. Bar=100 µm. (A_2_)-(G_2_) High magnification of the labyrinth. Bar =50 µm. # numbers and superscript numbers denote the placenta and the mouse identification number per experimental group shown in Table S2, respectively.

**Figure S3.** **Quantification of the number of *T. gondii* parasites in the placenta and uterus of dams.** The number of parasites in the placenta (A) and uterus (B) of the infected wild-type (WT) mice and the infected TLR2^-/^ mice at day 18.5 of pregnancy was measured by quantitative PCR. Each point in the graph represents one tissue, and the horizontal line indicates the mean value for each group. Statistical analysis was performed using the Mann-Whitney U test or t-test, but no difference was confirmed.

**Figure S4.** **Histopathological observations of placentas on day 18.5 of pregnancy (6 days postinfection).** Placenta were observed as tissue sections. The image on the left is an overall view of the placenta and the image on the right is a detail of the left figure. No differences were observed when 22 samples from infected wild-type (WT) mice were compared with 17 samples from uninfected WT mice. No differences were seen when 27 samples from infected TLR2^−/−^ mice were compared with 16 samples from uninfected TLR2^−/−^ mice. There were no morphological differences among the samples. Representative photographs are shown.
